# QTc interval prolongation and risk of atrial fibrillation recurrence: a meta-analysis and observational cohort study

**DOI:** 10.3389/fcvm.2024.1483591

**Published:** 2024-11-08

**Authors:** Qiuju Ding, Zhigang Wang, Lichong Lu, Zhizhao Song, Min Ge, Qing Zhou

**Affiliations:** Department of Cardio-Thoracic Surgery, Nanjing Drum Tower Hospital, The Affiliated Hospital of Nanjing University Medical School, Nanjing, China

**Keywords:** atrial fibrillation, catheter ablation, corrected QT interval, left atrial diameter, meta-analysis

## Abstract

**Introduction:**

Corrected QT interval (QTc) is a ventricular repolarization marker on electrocardiography. Previous studies evaluated its value in predicting atrial fibrillation (AF) occurrence. However, its predictive efficacy for AF recurrence remains controversial.

**Methods:**

We searched PubMed and Google databases for studies before January 2024 evaluating the association between QTc interval and AF incidence. A meta-analysis of the eligible datasets was conducted using Bazett's formula, with subgroup analysis to explore the heterogeneity. Additionally, thirty-eight patients with AF who underwent radiofrequency catheter ablation were enrolled and followed-up for 3–36 months. Univariate and multivariate Cox models were used to calculate the hazard ratios (HRs) and determine the relationship between clinical factors and AF recurrence. Kaplan-Meier survival analysis and ROC curve were conducted to assess the impact and predictive efficacy of individual factors.

**Results:**

Eleven datasets from nine eligible studies were enrolled and meta-analysed. We found that patients with prolonged QTc interval was associated with a significantly higher AF incidence risk, and the risk increased with every 10-ms prolongation. However, this association was not significant in the AF recurrence subgroup. In our prospective cohort, the preoperative body mass index, QTc, left atrial diameter (LAD), and uric acid levels influenced AF recurrence. Multivariate Cox regression analysis identified LAD as an independent factor affecting AF recurrence in patients with a high predictive efficiency. Kaplan-Meier survival analysis showed that increased LAD (>4.5 cm) was associated with postoperative AF recurrence.

**Discussion:**

Therefore, LAD has better predictive power and can be an indicator for predicting postoperative AF recurrence.

## Introduction

Atrial fibrillation (AF) is the most common sustained arrhythmia in the general population, with a morbidity rate of 1%–2% (1–3). It is a major cause of stroke, cardiac morbidity, and mortality, placing a heavy economic burden on society and families. The underlying mechanisms of AF are complex, including inflammation, atrial fibrosis, electrical remodelling, autonomic dysfunction, calcium-handling abnormalities, and oxidative stress. Although the exact aetiology of AF remains to be elucidated, it has been suggested that both structural and electrical remodelling are crucial to AF pathophysiology (4). Electrical abnormalities or structural endophenotypes, represented by electrocardiographic parameters, may play a role in the development of AF (5–8). A complex relationship has been suggested between AF and electrocardiographic parameters that reflect atrioventricular conduction (PR interval), ventricular depolarisation (QRS), ventricular repolarization [QT, QT corrected for heart rate (QTc), and JT interval], and cardiac contractions (RR interval and heart rate) (5–8).

The QT interval is obtained from a standard 12-lead electrocardiogram and is a ready, cheap, and fast measure of ventricular repolarization that is widely used as a predictor of life-threatening ventricular arrhythmias (9). Several studies have shown that a prolonged QT interval independently correlates with an increased risk of AF and stroke (10, 11). In a recent systematic review and meta-analysis, a prolonged QT interval was associated with an increased risk of AF (12). The association between QT prolongation and AF has been explained by abnormalities in myocardial repolarization (13). A prolonged QTc interval is a predictor of AF incidence; however, its role remains controversial (6, 12).

We, therefore, aimed to investigate whether prolonged QTc is a potential risk factor of AF, and to explore the real relationship between QTc interval extension and AF recurrence, providing a basis for precise treatment of different patients with AF.

## Results

### Prolonged corrected QT interval can predict AF incidence

**Figure 1:**
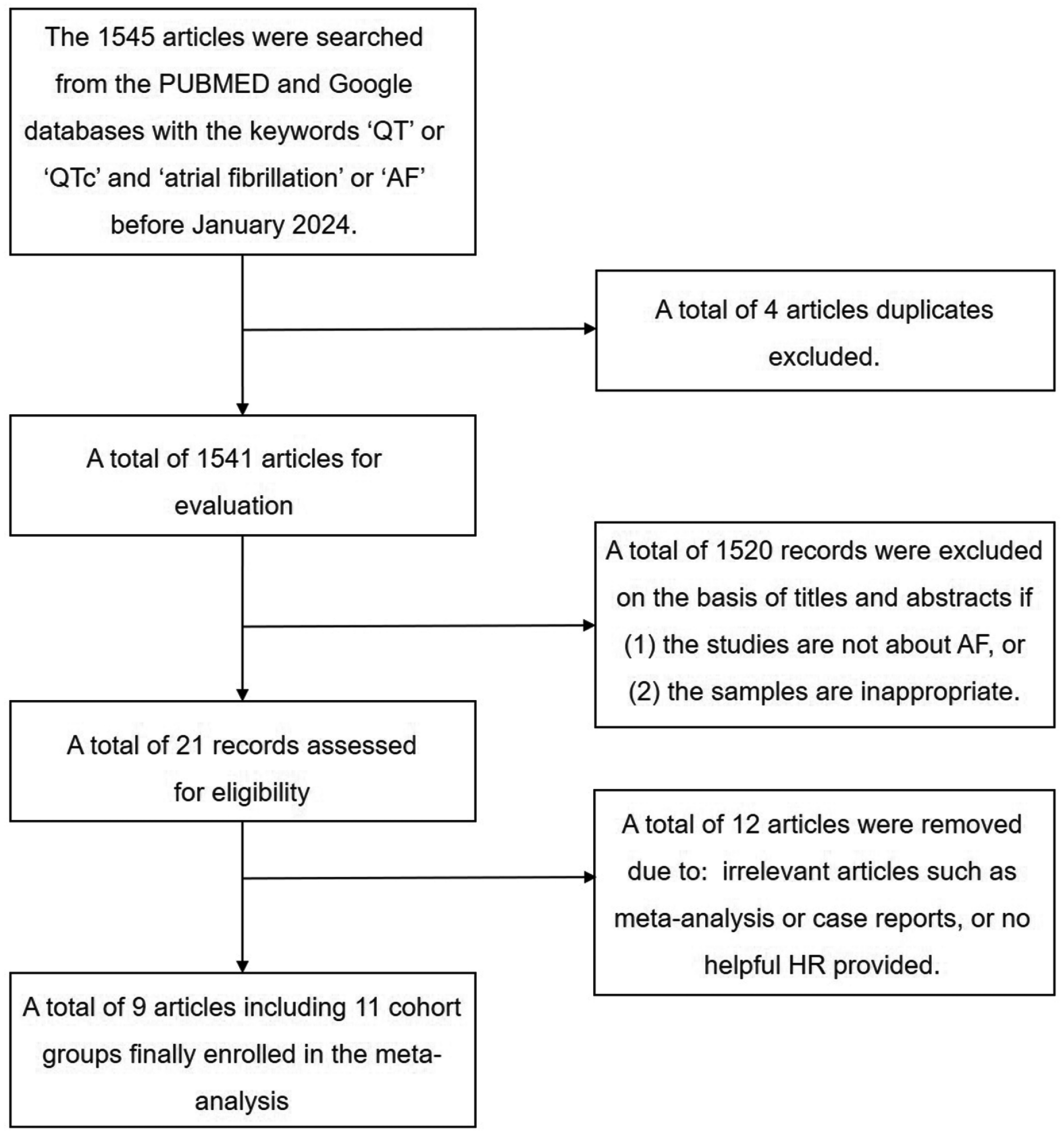
Flow chart of study selection process.

The study selection process is depicted in Figure 1. A total of 1,545 articles were initially included after searching the PubMed and Google databases. After removing four repetitive articles, we further excluded 1,520 records based on the title and abstract. Among the remaining 21 records, three articles investigated the same cohort studies included in another article with a larger sample size; two studies were meta-analyses, two studies were case reports, and five articles did not provide hazard ratios (HRs). Thus, nine articles, including 11 cohort groups with 338,801 patients aged 52–68 years, were included in the meta-analysis. Among the 11 cohort groups, 10 cohorts of the included studies (6, 9–11, 14–17) used a nonlinear regression formula (Bazett's) for QTc; thus, this method was used to analyse the pooled HRs in our meta-analysis. Six included groups from four studies used a linear regression formula to confirm robustness (6, 10, 11, 18). With regard to medications, only one study (11) kept a record of QTc interval-prolonging drugs, six studies (6, 9, 11, 15, 16, 18) listed medication use and three (10, 14, 17) did not mention medications. The detailed baseline characteristics of these studies are listed in Supplementary Table S1 online.

**Figure 2:**
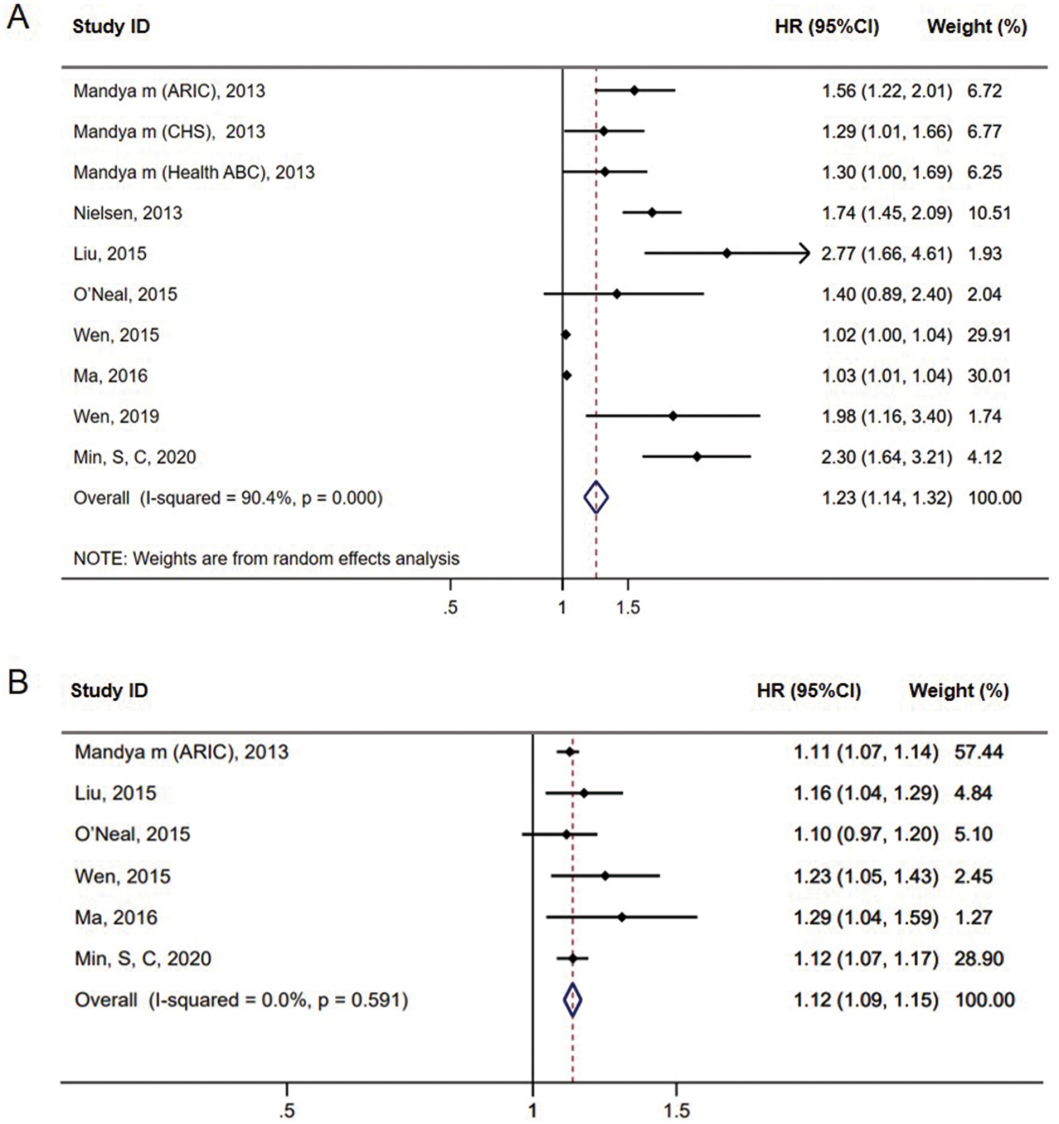
Forest plots of the association between prolonged QTc interval and AF incidence in meta-analysis based on Bazett's formula **(A)**. Forest plots of the pooled HRs for AF incidence every 10-ms prolongation in QTc interval based on Bazett's formula **(B)**.

Using the QTc as a categorical variable, our meta-analysis showed the QTc interval was associated with an increased risk of incident AF [HR = 1.23, 95% confidence Interval (CI) = 1.14–1.32, *P* < 0.01] based on Bazett's formula (Figure 2A). In the studies using linear regression formula to correct QT, the QTc interval was also associated with a higher risk of incident AF (HR = 1.67, 95% CI = 1.36–2.05, *P* < 0.01) (see Supplementary Figure S1 online). Furthermore, in the continuous variable analysis, we found a statistically significant risk of AF incidence for every 10-ms prolongation in QTc (HR = 1.12, 95% CI = 1.09–1.15, *P* < 0.01) (Figure 2B).

**Figure 3:**
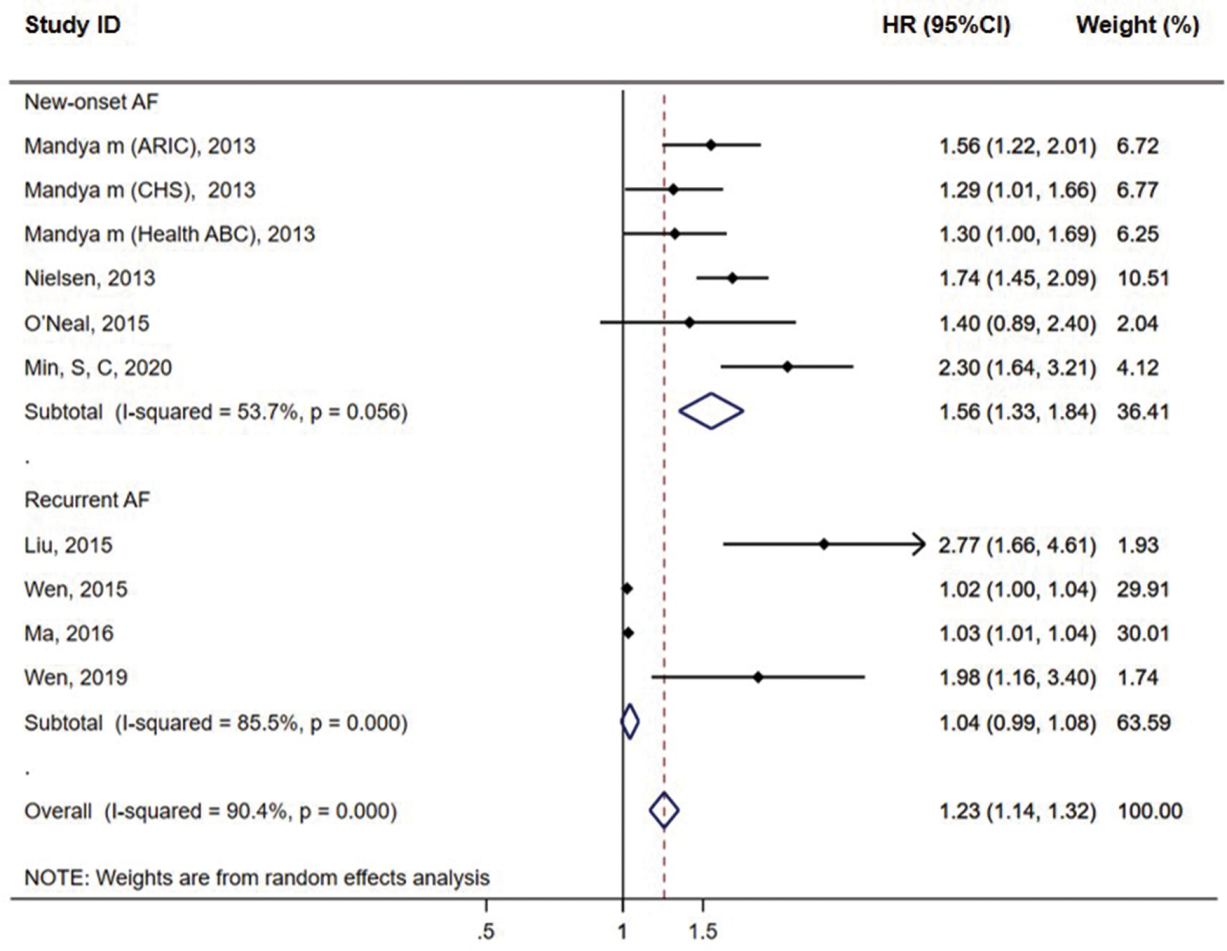
Subgroup analysis of the association between prolonged QTc interval and AF incidence in meta-analysis based on Bazett's formula.

Subgroup analysis showed that six cohort groups reporting the outcome of new-onset AF showed a significant association between QTc and AF risk (HR = 1.56, 95% CI = 1.33–1.84, *P* < 0.01) with the medium heterogeneity (*I*^2^ = 53.7%). For the remaining four groups reporting the recurrent AF, we found no association between QTc and AF risk (HR = 1.04, 95% CI = 0.99–1.08, *P* = 0.127) with significant heterogeneity (*I*^2^ = 85.5%) (Figure 3). No significant heterogeneity was observed in the studies with QTc cut-off values of ≥460 ms in female patients and ≥450 ms in male patients (*I*^2^ = 0%) and in the population from Europe (*I*^2^ = 24%), while significant heterogeneity was observed in the studies with mean age, follow-up duration of <5 years (*I*^2^ = 85.5%) and hospital-based study population (*I*^2^ = 85.5%) (see Supplementary Table S2 online). Publication bias was observed in the funnel plot based on Bazett's formula (see Supplementary Figure S2A online). However, publication bias was not evident in the studies based on linear regression formulae, which might be due to the small sample size (see Supplementary Figure S2B online).

### Prolonged corrected QT interval cannot predict AF recurrence

A total of 42 patients were enrolled in this prospective study. Nine patients were lost to follow-up and one patient died. Among 32 patients ultimately evaluated, the mean age was 60.91 ± 13.37 years, and the majority were male patients (78.13%). Notably, 14 patients had hypertension, 3 had diabetes, 11 had cardiovascular disease, 18 had persistent AF, 2 had received pacemaker implantation, and 23 patients received β blockers treatment. Warfarin was the most commonly used anticoagulant drug (53.13%). Only a minority of patients opted for the new oral anticoagulants, rivaroxaban and dabigatran. Ten patients were admitted with a history of smoking, whereas eight patients were admitted with a history of alcohol consumption. Among them, 14 developed AF recurrence after catheter ablation during follow-up. Compared with patients without AF recurrence, those with AF recurrence had a higher body mass index (BMI) (28.62 ± 3.96 vs. 24.17 ± 3.26, *P* = 0.002) and higher uric acid levels (455.57 ± 106.5 vs. 337.05 ± 87.27, *P* = 0.002). The usage of β-blockers was more common in patients with AF recurrence (92.86% vs. 55.56%, *P* = 0.02), and there was a higher proportion of patients with persistent AF (71.43% vs. 44.44%). The average left atrial diameter (LAD) was larger in patients with AF recurrence than in patients without with AF recurrence (4.89 ± 0.77 vs. 4.13 ± 0.33, *P* = 0.001), and the QTc interval was significantly prolonged among patients with AF recurrence (480.5 ± 43.82 vs. 445.28 ± 21.07, *P* = 0.0069). However, factors such as age, sex, smoking and alcohol history, laboratory tests (myocardial enzymes, liver enzymes, creatinine, and urea nitrogen), and the type of anticoagulant drug used had no significant relationship with recurrence (*P* > 0.05). Additionally, no significant difference was observed in terms of left heart ejection fraction or left ventricular diameter. Patient demographics and characteristics are summarised in Table 1.

**Table 1:** Demographic and clinical characteristics categorized by whether AF recurrent.

Characteristics	All (*n* = 32)	Recurrence (*n* = 14)	No recurrence (*n* = 18)	*P*-value
Age (Year)	60.91 ± 13.37	59.00 ± 13.04	62.39 ± 13.43	0.493
Male (%)	25 (78.13%)	11 (78.57%)	14 (77.78%)	0.957
BMI (kg/m^2^)	26.11 ± 4.20	28.62 ± 3.96	24.17 ± 3.26	0.002
Medications				
β-blocker (%)	23 (71.88%)	13 (92.86%)	10 (55.56%)	0.02
ACEU/ARB (%)	9 (28.13%)	6 (42.86%)	3 (16.67%)	0.102
Comorbidities				
HTN (%)	14 (43.75%)	6 (42.86%)	8 (44.44%)	0.928
DM (%)	3 (9.38%)	1 (7.14%)	2 (11.11%)	0.702
CVD (%)	11 (34.38%)	6 (42.86%)	5 (27.78%)	0.372
Type of AF				
Paroxysmal AF (%)	14 (43.75%)	4 (28.57%)	10 (55.56%)	0.127
Persistent AF (%)	18 (56.25%)	10 (71.43%)	8 (44.44%)	0.127
QTc (ms)	460.69 ± 37.35	480.50 ± 43.82	445.28 ± 21.07	0.007
Ultrasonic data				
LAD (cm)	4.46 ± 0.68	4.89 ± 0.77	4.13 ± 0.33	0.001
LVEF (%, IQR)	54.15 ± 8.20	52.09 ± 8.56	55.76 ± 7.52	0.221
LVDd (cm)	5.30 ± 0.84	5.49 ± 1.05	5.15 ± 0.60	0.271
LVDs (cm)	3.72 ± 0.81	3.90 ± 1.04	3.58 ± 0.53	0.295
Laboratory data				
CK (U/L)	521.00 ± 1646.40	1012.80 ± 2388.26	142.69 ± 281.11	0.227
CKMB (U/L)	16.13 ± 11.18	18.50 ± 12.27	14.31 ± 9.89	0.396
ALT (U/L)	32.66 ± 30.05	27.21 ± 12.71	36.92 ± 37.93	0.381
AST (U/L)	32.87 ± 34.18	36.98 ± 42.40	29.67 ± 25.59	0.563
Cystatin C (mg/L)	0.84 ± 0.89	0.85 ± 0.19	0.82 ± 0.18	0.697
SCr (umol/L)	73.13 ± 16.03	75.73 ± 13.88	70.94 ± 17.21	0.399
BUN (mmol/L)	6.58 ± 1.51	6.10 ± 1.49	6.96 ± 1.43	0.120
Uric acid (umol/L)	388.91 ± 112.71	455.57 ± 106.50	337.05 ± 87.27	0.002
Anticoagulation therapy				
Warfarin (%)	17 (53.13%)	7 (50%)	10 (55.56%)	0.754
Rivaroxaban (%)	1 (3.13%)	1 (7.14%)	0 (0%)	0.249
Dabigatran (%)	4 (12.5%)	2 (14.29%)	2 (11.11%)	0.788
Smoking (%)	10 (31.25%)	5 (35.71%)	5 (27.78%)	0.631
Drinking (%)	8 (25%)	5 (35.71%)	3 (16.67%)	0.217
Pacemaker implantation (%)	2 (6.25%)	0 (0%)	2 (11.11%)	0.198

Data are presented as *n* (%) and mean ± standard deviation.

AECI, angiotensin converting enzyme inhibitors; ALT, alanine transaminase; ARB, angiotensin II receptor blocker; AST, aspartate transaminase; BMI, body mass index; BUN, blood urea nitrogen; CI, confidence interval; CK, creatine kinase; CKMB, creatine kinase isoenzymes; CVD, cardiovascular diseases; DM, diabetes mellitus; LAD, left atrial diameter; HTN, hypertension; LVDd, left ventricular end diastolic dimension; LVDs, left ventricular end-systolic dimension; LVEF, left ventricular ejection fraction; QTc, corrected QT interval; sCr, serum creatinine.

**Figure 4:**
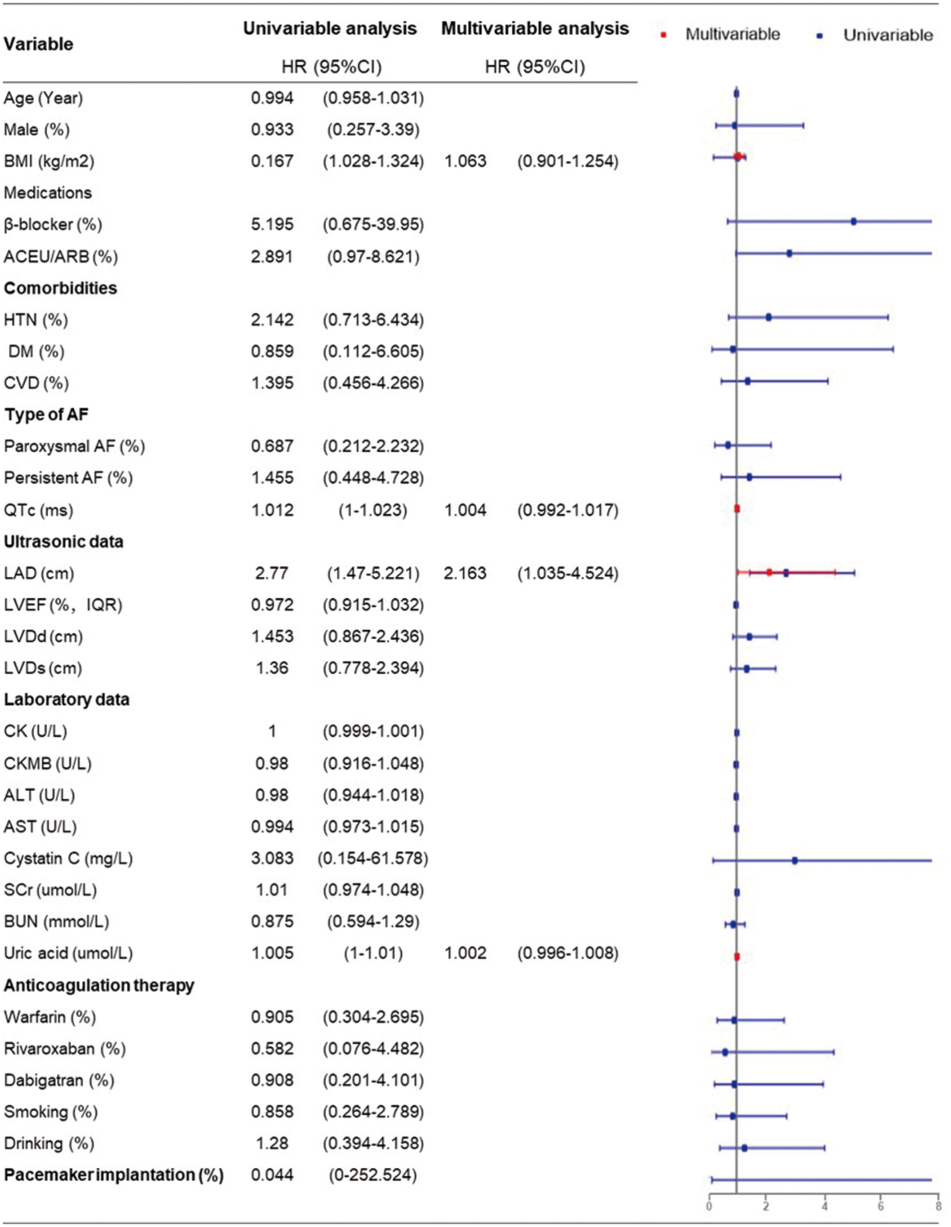
Univariable and multivariable cox regression analysis of risk factors for AF recurrence (multivariable cox regression analysis was performed including variables with *P*-value <0.1 by univariable analysis).

Univariate Cox regression analysis revealed that BMI (HR = 1.167, 95% CI = 1.028–1.324, *P* = 0.017), QTc (HR = 1.012, 95% CI = 1.00–1.023, *P* = 0.05), LAD (HR = 2.77, 95% CI = 1.47–5.221, *P* = 0.002), and uric acid level (HR = 1.005, 95% CI = 1.00–1.01, *P* = 0.046) were statistically associated with AF recurrence. Further multivariable Cox regression analysis was performed, including variables with a *P*-value < 0.1 by univariable analysis. This suggested that the LAD was an independent risk factor for the postoperative recurrence of AF (HR = 2.163, 95% CI = 1.035–4.524, *P* = 0.04) (Figure 4).

**Figure 5:**
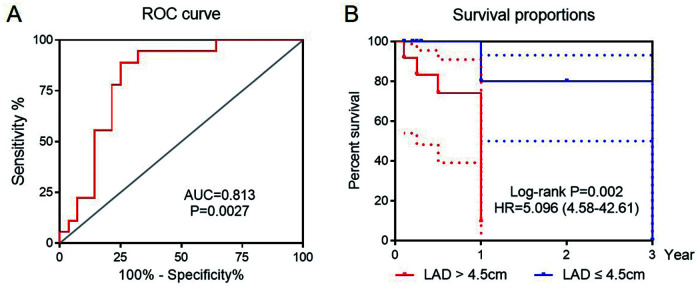
The receiver operating curve (ROC) for LAD prediction of AF recurrence after catheter ablation **(A)**. Kaplan Meier curve for sinus rhythm maintaining with LAD > 4.5 cm as the cut-off value **(B)**.

The area under the receiver operating characteristic curve (AUC) of preoperative LAD was 0.813 (95% CI = 0.645–0.982, *P* = 0.0027), while the AUC of QTc was 0.730 (95% CI = 0.538–0.922, *P* = 0.028). LAD had a better prediction threshold of 4.5 cm than that of QTc, with a sensitivity of 71.4% and a specificity of 88.9% (Figure 5A; see Supplementary Figure S4 online). The patients were divided into two groups based on the cut-off value 4.5 cm. The Kaplan–Meier curve showed that increased LAD was associated with postoperative AF recurrence (HR = 5.096, 95% CI = 4.58–42.61, log-rank *P* = 0.0002) (Figure 5B).

## Discussion

This comprehensive population-based cohort study offers a deeper understanding of the intricate link between QTc interval and AF. Our findings indicate a significant association between QTc interval and the onset of new AF cases in the general population yet no strong association with recurrent AF. Furthermore, during the follow-up period, we examined the association between recurrent AF and QTc while concurrently evaluating the clinical characteristics and laboratory test results. Finally, we demonstrated that pre-procedural LAD was associated with an elevated risk of recurrent AF. This study sheds light on the utility of echocardiography for identifying individuals who are more susceptible to developing AF in the future.

Underlying shared risk factors, including obesity, diabetes mellitus, coronary heart disease, and heart failure, can influence the cardiac conduction system. Additionally, these factors contribute to the development of AF (4), which is reflected by the prolongation of QT, QTc, and JT intervals on the electrocardiogram. Studies have indicated that a prolonged QTc interval is associated with the occurrence of AF and serves as a rapid, cost-effective, and valuable indicator for monitoring AF. This association has also been observed in patients with congenital long QT syndromes. A recent meta-analysis that pooled data from 309,676 individuals revealed a significant association between prolonged QTc interval and an increased risk of AF (12).

A longer QT interval may directly reflect a greater propensity for AF, as it may be a manifestation of aberrations in refractoriness that occur simultaneously in both the atrium and ventricle. For instance, patients with long QT Syndrome who have prolonged cardiomyocyte refractoriness often experience polymorphic atrial tachycardia characterised by an undulating *P*-wave axis. This condition, known as “atrial torsade de pointes” (19), has been shown to potentially progress into AF (20). Another plausible explanation is the augmented activity of the late sodium current. An elevated influx of late sodium into cardiomyocytes is reflected in the 12-lead electrocardiogram by QT interval prolongation (21, 22) and by augmented intracellular calcium levels, ultimately triggering automaticity. This cascade of events may facilitate the onset of AF (23, 24).

Catheter ablation is a commonly used first-line treatment for patients with symptomatic AF recurrence despite anti-arrhythmic drug therapy. However, the recurrence rate remains high, with over half of patients requiring multiple ablation procedures (25). Our meta-analysis confirmed that QTc can predict the initial occurrence of AF but failed to detect a statistically significant correlation between QTc levels and AF recurrence. The sensitivity analysis showed that after excluding the study by Liu et al. (9), the association result became significant (*P value* = 0.043, see Supplementary Table S3 online). Thus, we speculated that the reason for the non-significant result may be the different study type by Liu et al. Moreover, although we used a random effects model to account for heterogeneity and improve the estimate robustness, our initial meta-analysis showed high heterogeneity (*I*^2^ = 90.4%). To explore its sources, we conducted subgroup analyses based on AF type, cut-off time, mean age, population, follow-up duration, and patient source. For the new-onset AF group with consistent follow-up duration, patient sources, and study type, the moderate heterogeneity (*I*^2^ = 53.7%) may have resulted from differences in the race, mean age, cut-off time, and medication use. For the recurrent AF group with consistent follow-up duration and patient sources, although differences in the race, mean age, cut-off time, and medication use also existed, high heterogeneity (*I*^2^ = 85.5%) persisted, possibly due to the inconsistency in the design in Liu et al.'s study in 2015.

Notably, there remains some controversy and uncertainty surrounding the research results regarding QTc prolongation as a predictor of AF (6). Some researchers have proposed that the LAD has higher predictive power for AF than that of QTc prolongation, whereas some have argued that the combined use of LAD and QTc is superior to the use of either parameter alone in predicting AF recurrence among patients with hypertrophic cardiomyopathy (HCM) following ablation therapy (14).

Left atrial (LA) enlargement results from atrial remodelling, impacting LA function and potentially promoting electrical remodelling, which exacerbates AF. It is defined as anterior–posterior LAD ≥ 4.0 cm and has been significantly associated with incident AF, stroke, and death; it is also considered a key risk factors for the development of AF (26). Current guidelines recommend intensification of diagnostic arrhythmia surveillance with 48 h Holter monitoring at 6-month intervals once LAD is ≥4.5 cm (27). However, over 50% of HCM patients develop new-onset AF despite preserved baseline LAD of <4.5 cm (28). Furthermore, LAD ≥ 5.5 cm is associated with poor survival (29). In the Cardiovascular Health Study, a 10-mm increment in the anteroposterior LAD was associated with a 74% increased risk of new-onset AF (30).

Consistent with prior reports, the LAD artery emerged as an independent predictor of arrhythmia recurrence following catheter ablation in the present study (HR = 2.77), and the cut-off value of LAD was 4.5 cm, indicating that the patients with preoperative increased LAD have a nearly 1.7-fold increased risk of AF recurrence compared to normal individuals, with a statistical significance (*P* = 0.002). Notably, when LAD exceeds 4.5 cm, recurrence risk increases approximately four times (HR = 5.096, *P* = 0.0002). Thus, for such patients, ablation surgery should be cautiously considered. For those already treated with radiofrequency ablation, LAD should be monitored to assess surgical outcomes and recurrence risk, which can aid in personalized treatment planning.

Although there is an ongoing debate regarding the independent predictive value of a dilated LA for post-ablation AF recurrence, several plausible theories have been proposed. First, chronic dilation of the LA strongly promotes structural remodelling and vice versa. More recently, some experts suggested that patients with moderate LA dilation who do not exhibit reduced LA compliance respond favourably to the reversal of LA remodelling following ablation. However, patients with more advanced remodelling of the LA may not respond favourably to this treatment (31, 32). Although the mechanisms underlying structural remodelling are highly intricate, the primary changes observed in the atrium manifest as decreases in the myocardium and increases in atrial fibrosis (33). Atrial fibrosis, a signature characteristic of atrial structural remodelling, triggers and perpetuates AF by altering the substrate of the LA and subsequently inducing electrical remodelling (34). Second, it is conceivable that patients with an enlarged LA may require more energy and longer lesions to achieve complete ablation. Several studies have demonstrated that severe LA scarring post-ablation predisposes patients to recurrent AF, which seems to be due to re-conduction between the LA and pulmonary veins (PVs) (35). Simultaneously, the presence of pre-existing LA scarring alongside a dilated LA has unfavourable implications for atrial fibrosis and may potentially reduce the success rate following ablation (36). These findings may explain why extensive ablation or re-ablation does not significantly improve the success rate in patients with extensive LA scarring. Perhaps, routine repeat ablation sessions are not necessary for patients with severe LA enlargement or scarring.

The key strengths of the present study are its population-based approach, extensive sample size, and rigorous verification of AF events. However, our study also has some limitations that need to be considered. For the meta-analysis, the majority of the studies included were observational, indicating that the results may have been influenced by unmeasured or residual confounding factors. Furthermore, the association between prolonged QTc interval and AF recurrence was not significant in our meta-analysis, which may be attributed to the variability in sample sizes and different study types among the included studies. Additionally, it is challenging to eliminate the potential impact of QTc interval-prolonging drugs on baseline QTc because few studies provided details on the utilization of these drugs. Moreover, the high heterogeneity observed in the recurrence subgroups limits the validity of our study conclusions. Future large-sample, multi-ethnic meta-analysis should be more rigorous in the selection of patients and research methods. In the prospective cohort, we defined the blanking period as 3 months, which is commonly accepted within the current field (37, 38). Considering that different blanking periods can lead to different results in terms of QTc prolongation (39), future studies are needed to further refine the definition of the blanking period and consider multiple blanking period settings to evaluate the impact on QTc prolongation. We could not differentiate between paroxysmal, persistent, and permanent AF, given that Holter monitoring was not conducted in this population-based cohort. Thus, the presence of asymptomatic or paroxysmal AF may lead to underestimation of the recurrence rate. An association between QTc and recurrent AF was not detected because of the limited number of patients undergoing transcatheter ablation. Our prospective cohort primarily comprised a limited sample size, focusing on older individuals of Chinese descent. In the future, we intend to collaborate with multiple centres, both domestically and internationally, to further increase the sample size and patient diversity, thereby enhancing the statistical power and generalizability of our findings.

In summary, our meta-analysis indicates that QTc serves as a rapid, cost-effective, and valuable indicator of AF. However, it lacks predictive capability for postoperative AF recurrence, whereas LAD can independently predict postoperative AF recurrence. Future large-scale prospective studies are necessary to elucidate the potential electrophysiological mechanisms underlying LAD's association with increased AF recurrence.

## Methods

### Meta-analysis

We searched the PubMed and Google database systematically using the key words “QT” or “QTc” and “atrial fibrillation” or “AF” from inception to January 2024. The exclusion criteria were (1) repetitive studies, (2) inappropriate samples, (3) lack of a case-control study design, (4) not related to AF, or (5) no helpful HR provided. The inclusion criteria were as follows: (1) prospective or retrospective cohort and case-control studies; (2) research on the relationship between QTc interval and AF; (3) new-onset or recurrent AF as an endpoint; (4) HR and corresponding 95% CI as the effect size; (5) published English language articles; and (6) human participants. As depicted in Figure 1, two independent investigators (Q.D. and Z.W.) reviewed all titles or abstracts and then conducted an elaborative screening of all articles based on full-text. The baseline characteristics of each study were independently extracted by two independent reviewers (Q.D and Z.W). In case of a disagreement, the senior author (Q.Z.) was consulted to reach agreement.

The methods employed to adjust the QT interval based on heart rate, as well as the cut-off values of the QTc interval utilised in these studies, were documented. Bazett's formula is a one of correction methods for dividing QT interval by the heart rate, i.e.,: QTc = QT/RR^1/2^, and linear regression formulae consist of Framingham linear regression formula [QTc = QT + 0.154 (1-RR)], as well as Hodges’ formula [QTc = QT + 0.00175 (HR −60)]. HRs and their corresponding 95% CIs were calculated to assess the impact of the QTc interval on AF risk. The primary endpoints of the articles included in this meta-analysis were new-onset and recurrent AF in clinical settings. Additionally, relevant data were gathered to explore the association between a 10-ms prolongation of the QTc interval and the risk of AF. If multiple articles were derived from the same cohort, we prioritized selecting the article that provided the most comprehensive reporting of the QTc interval or exhibited the largest sample size. The baseline characteristics of each study are presented in Supplementary Table S1 online.

In a comprehensive meta-analysis, the pooled HRs and corresponding 95% CIs between prolonged QTc interval and AF risk were estimated based on Bazett's or linear regression formulae in the categorical variable analysis. Tests of heterogeneity of the HRs across studies were estimated using the chi-square and I-square, with fixed-effects models or random effects models based on the criteria of *P* > 0.10 and *I*^2^ <50%. In the continuous variable analysis, the association between QTc every 10-ms prolongation and AF risk was assessed. Subgroup analysis was performed to explore the origin of heterogeneity in this meta-analysis. Funnel plots were used to assess publication bias, and sensitivity analyses were applied to measure the influence of each study. A two-tailed *P* value < 0.05 was considered statistically significant. All data were assessed using Stata (Version 17.0; Stata Corp., College Station, TX, USA).

### Prospective study cohort

#### Patients

This prospective observational study enrolled 42 consecutive patients with paroxysmal or persistent AF, who underwent radiofrequency catheter ablation (RFCA) at our institution between January 2020 and December 2022. The inclusion criteria were as follows: age > 18 years, non-valvular AF, suitability for catheter ablation, voluntary participation in the study, and signed informed consent. The exclusion criteria were as follows: New York Heart Association functional classes III and IV; progressive renal insufficiency ≤ 3 months before ablation; myocardial infarction or percutaneous coronary intervention ≤ 6 months before ablation; presence of LA thrombus on transoesophageal echocardiography; and the presence of a prosthetic heart valve. Patients with a malignancy, autoimmune or inflammatory disease, or severe hepatic or renal dysfunction before ablation were excluded. The Ethics Committee of Nanjing University approved this study (Ethical Number: 2021-632-02), and all participants provided written informed consent.

#### Catheter ablation

All patients discontinued their anti-arrhythmic drugs for at least 3 months prior to RFCA. Transoesophageal echocardiography was performed 1 day before the procedure to confirm the absence of atrial thrombi. RF-based circumferential pulmonary vein (PV) isolation was performed using an irrigated RF ablation catheter (Thermocool; Biosense Webster, Diamond Bar, CA, USA) facilitated by electroanatomic mapping (Carto 3; Biosense Webster). Complete pulmonary vein isolation was achieved in all the patients. Additional ablation procedures such as superior vena cava isolation, linear ablation of the left atrial roof, and cavotricuspid isthmus ablation were performed at the operator's discretion. Successful ablation was defined as the complete elimination of fragmented signals at the PV ostium, along with the confirmation of both exit and entrance blocks. If AF or other types of sustained atrial tachyarrhythmia persisted after the initial ablation, direct current cardioversion was performed to restore sinus rhythm.

#### Follow up and clinical data collection

Patients were evaluated every 3 months in the dedicated arrhythmia outpatient clinic for ≥12 months. Routine echocardiograms and electrocardiograms were obtained at each outpatient visit to evaluate AF recurrence. AF recurrence was defined as any documented atrial arrhythmia (AF, atrial flutter, or atrial tachycardia) episode lasting > 30 s after ablation, excluding a 3-month blanking period. Patient information including demographics, medical histories, results of physical examinations and laboratory tests, imaging findings, treatments, and outcomes were retrieved from the hospital's electronic database.

#### Statistical analysis

Continuous variables are presented as means ± standard deviation, and differences were determined using the one-way ANOVA or Student's *t*-test for normally distributed data. Non-normally distributed data are presented as median (inter-quartile range), and differences were assessed using the Mann–Whitney *U*-test. Categorical data are presented as *n* (%) using the chi-square or Fisher's exact test. Univariate and multivariate Cox proportional hazards models were used to calculate the HRs and determine the relationship between clinical factors and AF recurrence. Kaplan–Meier survival analysis was conducted to calculate the impact of individual factors on AF recurrence. The predictive efficacy of this factor was evaluated using an ROC curve. Schoenfeld residuals were utilized to validate proportional hazards, and the linearity assumption for continuous variables was checked by Martingale residuals. Each Cox regression model satisfied all the underlying assumptions (see Supplementary Figure S3 online). The *P*-value of <0.05 was considered statistically significant. All statistical analyses were performed using SPSS version 23 software (SPSS Inc., Chicago, IL, USA).

## Data Availability

The original contributions presented in the study are included in the article/Supplementary Material, further inquiries can be directed to the corresponding author.

## References

[1] Cheng M Lu X Huang J Zhang S Gu D. Electrocardiographic PR prolongation and atrial fibrillation risk: a meta-analysis of prospective cohort studies. J Cardiovasc Electrophysiol. (2015) 26(1):36–41. 10.1111/jce.12539 25199533

[2] Go AS Hylek EM Phillips KA Chang Y Henault LE Selby JV Prevalence of diagnosed atrial fibrillation in adults: national implications for rhythm management and stroke prevention: the AnTicoagulation and risk factors in atrial fibrillation (ATRIA) study. JAMA. (2001) 285(18):2370–5. 10.1001/jama.285.18.2370 11343485

[3] January CT Wann LS Alpert JS Calkins H Cigarroa JE Cleveland JC Jr. 2014 AHA/ACC/HRS guideline for the management of patients with atrial fibrillation: a report of the American college of cardiology/American heart association task force on practice guidelines and the heart rhythm society. J Am Coll Cardiol. (2014) 64(21):e1–76. 10.1016/j.jacc.2014.03.022 24685669

[4] Hindricks G Potpara T Dagres N Arbelo E Bax JJ Blomström-Lundqvist C 2020 ESC guidelines for the diagnosis and management of atrial fibrillation developed in collaboration with the European association for cardio-thoracic surgery (EACTS): the task force for the diagnosis and management of atrial fibrillation of the European society of cardiology (ESC) developed with the special contribution of the European heart rhythm association (EHRA) of the ESC. Eur Heart J. (2021) 42(5):373–498. 10.1093/eurheartj/ehaa612 32860505

[5] Aeschbacher S O'Neal WT Krisai P Loehr L Chen LY Alonso A Relationship between QRS duration and incident atrial fibrillation. Int J Cardiol. (2018) 266:84–8. 10.1016/j.ijcard.2018.03.050 29887479 PMC6027639

[6] O'Neal WT Efird JT Kamel H Nazarian S Alonso A Heckbert SR The association of the QT interval with atrial fibrillation and stroke: the multi-ethnic study of atherosclerosis. Clin Res Cardiol. (2015) 104(9):743–50. 10.1007/s00392-015-0838-z 25752461 PMC4945099

[7] Schumacher K Dagres N Hindricks G Husser D Bollmann A Kornej J. Characteristics of PR interval as predictor for atrial fibrillation: association with biomarkers and outcomes. Clin Res Cardiol. (2017) 106(10):767–75. 10.1007/s00392-017-1109-y 28382425

[8] Smith JW O'Neal WT Shoemaker MB Chen LY Alonso A Whalen SP PR-Interval Components and atrial fibrillation risk (from the atherosclerosis risk in communities study). Am J Cardiol. (2017) 119(3):466–72. 10.1016/j.amjcard.2016.10.016 27889043 PMC5531862

[9] Liu N Wen SN Ruan Y Zhang T Li SN Wu JH QTc interval prolongation predicts the ablation outcome in hypertensive patients with paroxysmal atrial fibrillation. Eur Heart J Suppl. (2015) 17(suppl_B):B32–B8. 10.1093/eurheartj/suv017

[10] Mandyam MC Soliman EZ Alonso A Dewland TA Heckbert SR Vittinghoff E The QT interval and risk of incident atrial fibrillation. Heart Rhythm. (2013) 10(10):1562–8. 10.1016/j.hrthm.2013.07.023 23872693 PMC3787974

[11] Nielsen JB Graff C Pietersen A Lind B Struijk JJ Olesen MS J-shaped association between QTc interval duration and the risk of atrial fibrillation: results from the Copenhagen ECG study. J Am Coll Cardiol. (2013) 61(25):2557–64. 10.1016/j.jacc.2013.03.032 23583581

[12] Zhang N Gong M Tse G Zhang Z Meng L Yan BP Prolonged corrected QT interval in predicting atrial fibrillation: a systematic review and meta-analysis. Pacing Clin Electrophysiol. (2018) 41(3):321–7. 10.1111/pace.13292 29380395

[13] Johnson JN Tester DJ Perry J Salisbury BA Reed CR Ackerman MJ. Prevalence of early-onset atrial fibrillation in congenital long QT syndrome. Heart Rhythm. (2008) 5(5):704–9. 10.1016/j.hrthm.2008.02.007 18452873 PMC3940082

[14] Wen SN Liu N Li SN Wu XY Salim M Kang JP QTc interval prolongation predicts arrhythmia recurrence after catheter ablation of atrial fibrillation in patients with hypertrophic cardiomyopathy. Circ J. (2015) 79(5):1024–30. 10.1253/circj.CJ-14-1290 25739859

[15] Ma N Wu XY Ma CS Liu N Bai R Du X QTc interval predicts outcome of catheter ablation in paroxysmal atrial fibrillation patients with type 2 diabetes mellitus. J Huazhong Univ Sci Technol Med Sci. (2016) 36(5):646–52. 10.1007/s11596-016-1640-5 27752887

[16] Wen SN Zhu HJ Sun PY Wu K Liu N Ruan YF Depolarization and repolarization parameters on ECG predict recurrence after atrial fibrillation ablation in patients with hypertrophic cardiomyopathy. J Cardiovasc Electrophysiol. (2019) 30(11):2405–13. 10.1111/jce.14137 31441155

[17] Cho MS Nam GB Kim YN Kim J Choi KJ Kim YH. Clinical implications of ventricular repolarization parameters on long-term risk of atrial fibrillation- longitudinal follow-up data from a general ambulatory Korean population. Circ J. (2020) 84(7):1067–74. 10.1253/circj.CJ-19-1151 32461513

[18] Geurts S Tilly MJ Kors JA Deckers JW Stricker BHC de Groot NMS Electrocardiographic parameters and the risk of new-onset atrial fibrillation in the general population: the rotterdam study. Europace. (2023) 25(6). 10.1093/europace/euad164 PMC10299895 37369558

[19] Kirchhof P Eckardt L Franz MR Mönnig G Loh P Wedekind H Prolonged atrial action potential durations and polymorphic atrial tachyarrhythmias in patients with long QT syndrome. J Cardiovasc Electrophysiol. (2003) 14(10):1027–33. 10.1046/j.1540-8167.2003.03165.x 14521653

[20] Satoh T Zipes DP. Cesium-induced atrial tachycardia degenerating into atrial fibrillation in dogs: atrial torsades de pointes? J Cardiovasc Electrophysiol. (1998) 9(9):970–5. 10.1111/j.1540-8167.1998.tb00137.x 9786077

[21] Lowe JS Stroud DM Yang T Hall L Atack TC Roden DM. Increased late sodium current contributes to long QT-related arrhythmia susceptibility in female mice. Cardiovasc Res. (2012) 95(3):300–7. 10.1093/cvr/cvs160 22562703 PMC3633400

[22] Zygmunt AC Eddlestone GT Thomas GP Nesterenko VV Antzelevitch C. Larger late sodium conductance in M cells contributes to electrical heterogeneity in canine ventricle. Am J Physiol Heart Circ Physiol. (2001) 281(2):H689–97. 10.1152/ajpheart.2001.281.2.H689 11454573

[23] Song Y Shryock JC Belardinelli L. An increase of late sodium current induces delayed afterdepolarizations and sustained triggered activity in atrial myocytes. Am J Physiol Heart Circ Physiol. (2008) 294(5):H2031–9. 10.1152/ajpheart.01357.2007 18310511

[24] Voigt N Li N Wang Q Wang W Trafford AW Abu-Taha I Enhanced sarcoplasmic reticulum Ca2+ leak and increased Na+-Ca2+ exchanger function underlie delayed afterdepolarizations in patients with chronic atrial fibrillation. Circulation. (2012) 125(17):2059–70. 10.1161/CIRCULATIONAHA.111.067306 22456474 PMC4663993

[25] Imberti JF Ding WY Kotalczyk A Zhang J Boriani G Lip G Catheter ablation as first-line treatment for paroxysmal atrial fibrillation: a systematic review and meta-analysis. Heart (Bri Cardiac Soc). (2021) 107(20):1630–6. 10.1136/heartjnl-2021-319496 34261737

[26] Conen D Glynn RJ Sandhu RK Tedrow UB Albert CM. Risk factors for incident atrial fibrillation with and without left atrial enlargement in women. Int J Cardiol. (2013) 168(3):1894–9. 10.1016/j.ijcard.2012.12.060 23333369 PMC3643987

[27] Elliott PM Anastasakis A Borger MA Borggrefe M Cecchi F Charron P 2014 ESC guidelines on diagnosis and management of hypertrophic cardiomyopathy: the task force for the diagnosis and management of hypertrophic cardiomyopathy of the European society of cardiology (ESC). Eur Heart J. (2014) 35(39):2733–79. 10.1093/eurheartj/ehu284 25173338

[28] Debonnaire P Joyce E Hiemstra Y Mertens BJ Atsma DE Schalij MJ Left atrial size and function in hypertrophic cardiomyopathy patients and risk of new-onset atrial fibrillation. Circ Arrhythm Electrophysiol. (2017) 10(2). 10.1161/CIRCEP.116.004052 28183843

[29] Rusinaru D Tribouilloy C Grigioni F Avierinos JF Suri RM Barbieri A Left atrial size is a potent predictor of mortality in mitral regurgitation due to flail leaflets: results from a large international multicenter study. Circ Cardiovasc Imaging. (2011) 4(5):473–81. 10.1161/CIRCIMAGING.110.961011 21737598

[30] Psaty BM Manolio TA Kuller LH Kronmal RA Cushman M Fried LP Incidence of and risk factors for atrial fibrillation in older adults. Circulation. (1997) 96(7):2455–61. 10.1161/01.CIR.96.7.2455 9337224

[31] Kuppahally SS Akoum N Badger TJ Burgon NS Haslam T Kholmovski E Echocardiographic left atrial reverse remodeling after catheter ablation of atrial fibrillation is predicted by preablation delayed enhancement of left atrium by magnetic resonance imaging. Am Heart J. (2010) 160(5):877–84. 10.1016/j.ahj.2010.07.003 21095275 PMC2995281

[32] Tops LF Delgado V Bertini M Marsan NA Den Uijl DW Trines SA Left atrial strain predicts reverse remodeling after catheter ablation for atrial fibrillation. J Am Coll Cardiol. (2011) 57(3):324–31. 10.1016/j.jacc.2010.05.063 21232671

[33] Burstein B Nattel S. Atrial fibrosis: mechanisms and clinical relevance in atrial fibrillation. J Am Coll Cardiol. (2008) 51(8):802–9. 10.1016/j.jacc.2007.09.064 18294563

[34] Yue L Xie J Nattel S. Molecular determinants of cardiac fibroblast electrical function and therapeutic implications for atrial fibrillation. Cardiovasc Res. (2011) 89(4):744–53. 10.1093/cvr/cvq329 20962103 PMC3039247

[35] Oakes RS Badger TJ Kholmovski EG Akoum N Burgon NS Fish EN Detection and quantification of left atrial structural remodeling with delayed-enhancement magnetic resonance imaging in patients with atrial fibrillation. Circulation. (2009) 119(13):1758–67. 10.1161/CIRCULATIONAHA.108.811877 19307477 PMC2725019

[36] Verma A Wazni OM Marrouche NF Martin DO Kilicaslan F Minor S Pre-existent left atrial scarring in patients undergoing pulmonary vein antrum isolation: an independent predictor of procedural failure. J Am Coll Cardiol. (2005) 45(2):285–92. 10.1016/j.jacc.2004.10.035 15653029

[37] Calkins H Gache L Frame D Boo LM Ghaly N Schilling R Predictive value of atrial fibrillation during the postradiofrequency ablation blanking period. Heart Rhythm. (2021) 18(3):366–73. 10.1016/j.hrthm.2020.11.020 33242668

[38] Themistoclakis S Schweikert RA Saliba WI Bonso A Rossillo A Bader G Clinical predictors and relationship between early and late atrial tachyarrhythmias after pulmonary vein antrum isolation. Heart Rhythm. (2008) 5(5):679–85. 10.1016/j.hrthm.2008.01.031 18325850

[39] Mohanty S Torlapati PG Casella M Della Rocca DG Schiavone M Doty B Redefining the blanking period after pulsed-field ablation in patients with atrial fibrillation. Heart Rhythm. (2024). 10.1016/j.hrthm.2024.08.011 39117003

