# Supplementary material for: QTc interval prolongation and risk of atrial fibrillation recurrence: a meta-analysis and observational cohort study
Source: Front Cardiovasc Med. 2024 Nov 8;11:1483591. doi: 10.3389/fcvm.2024.1483591 (PMC11582060; doi:10.3389/fcvm.2024.1483591)
Supplement: Supplementary file 1 [file Datasheet1.docx]

**QTc interval prolongation and risk of atrial fibrillation recurrence:**

**a meta-analysis and observational cohort study**

**Supplementary Fig. S1** Forest plots of the association between prolonged QTc interval and AF incidence in meta-analysis based on linear regression formulae.


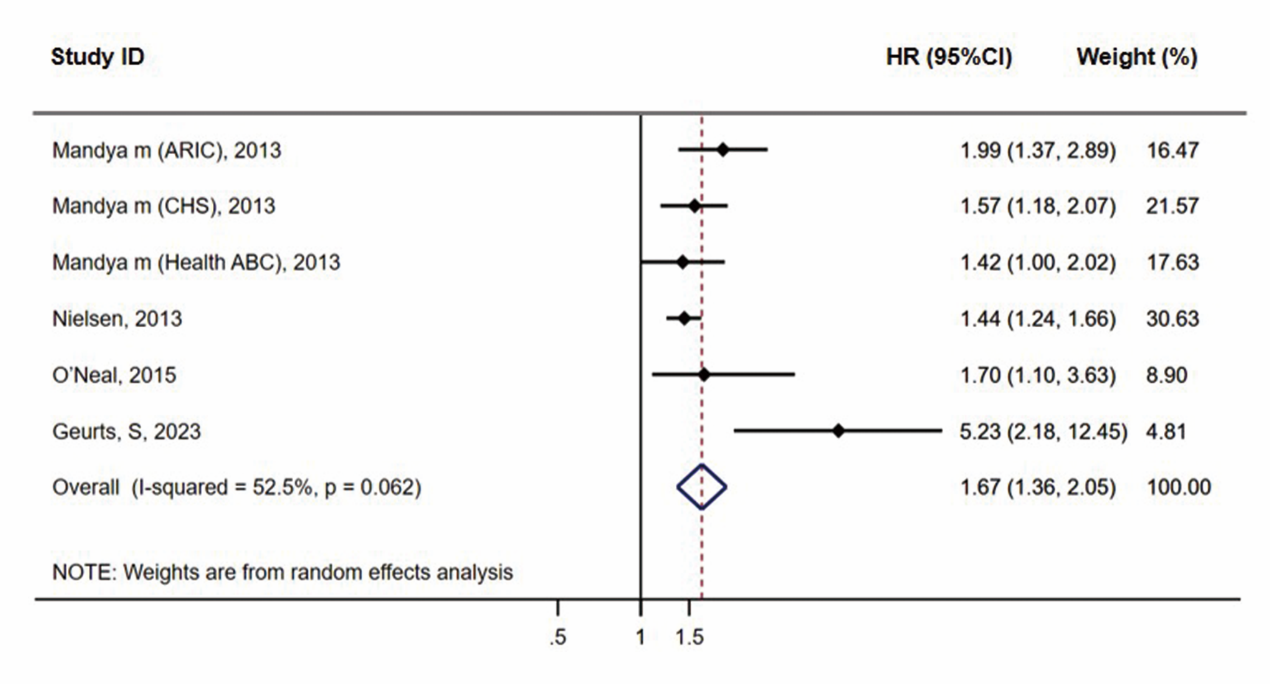


**Supplementary Fig. S2** Funnel bias of the included studies. (A) Studies analyzed based on Bazett’s formula; (B) Studies analyzed based on linear regression formulae.


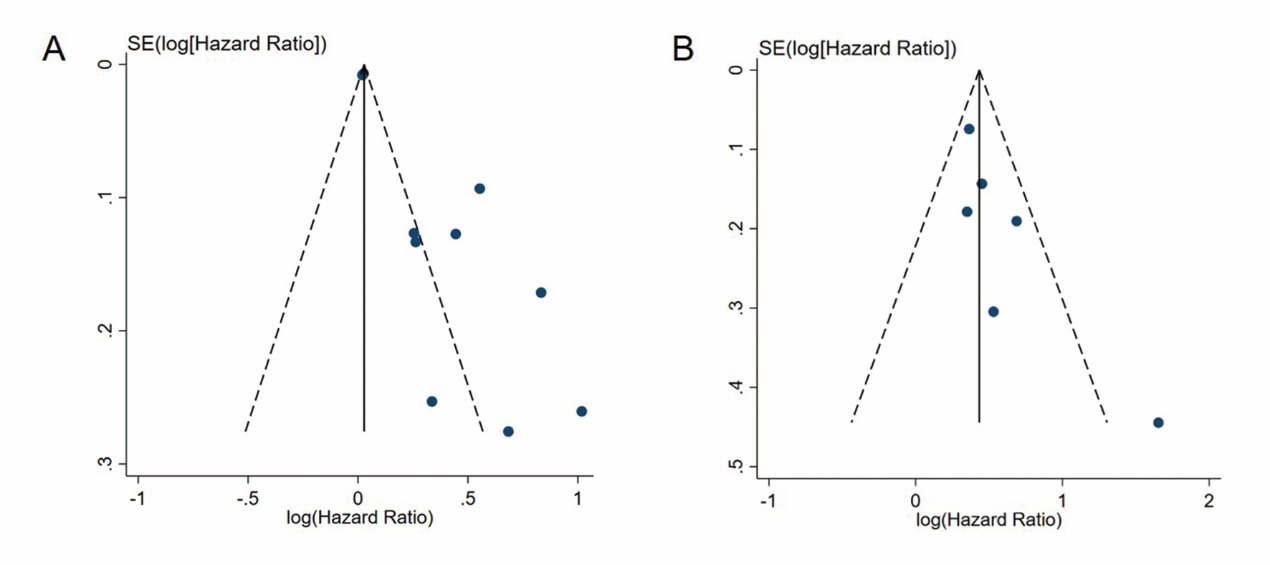


**Supplementary Fig. S3** (A) Time-varying Schoenfeld residuals and (B) Martingale residuals in Cox regression models.


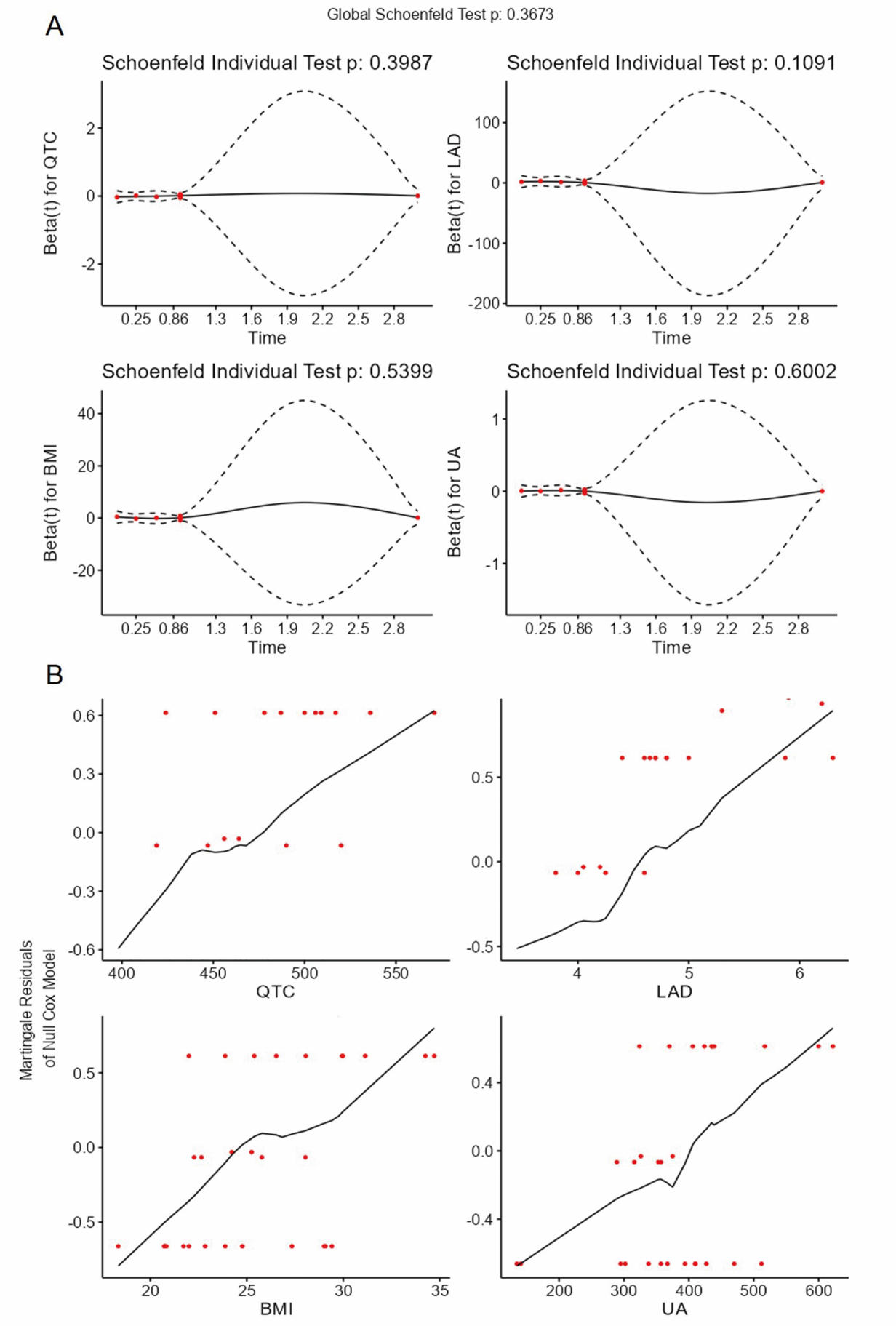


**Supplementary Fig.S4** The Receiver Operating Curve (ROC) for LAD (A) and QTc (B) prediction of AF recurrence.


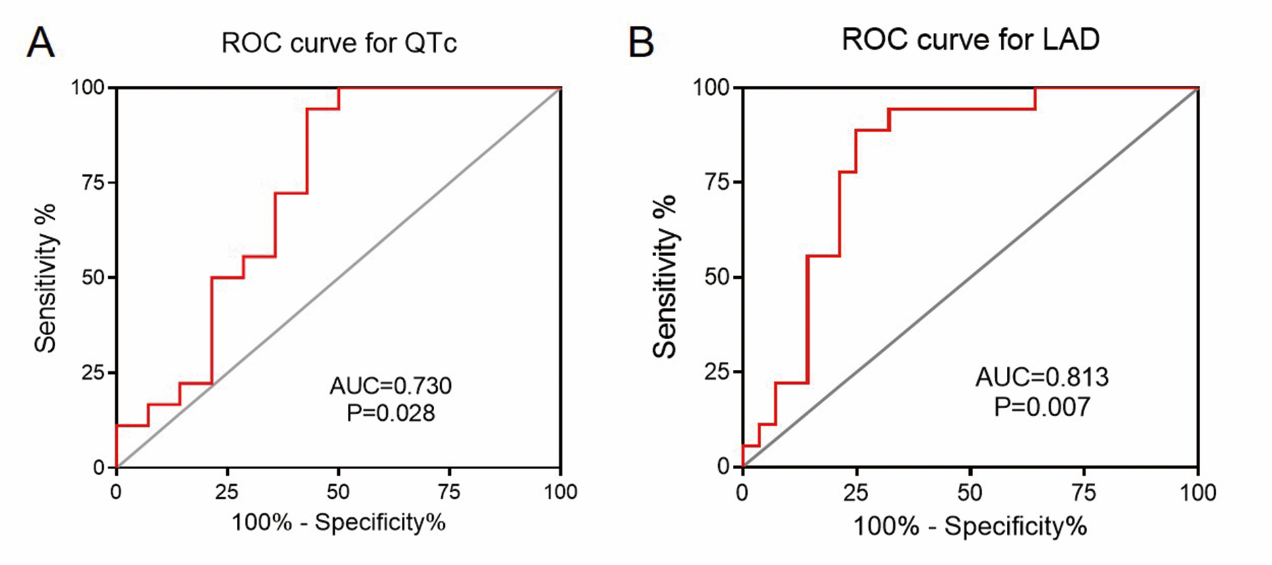


**Supplementary Table S1** Characteristics of the 11 cohorts from 9 articles included after screening for this meta-analysis.

| **Cohorts, Year** | **Study design** | **Study location** | **Population** | **Samples, n** | **Study population** | **Definition of prolonged QTc** | **End point** | **Follow-up** |
| --- | --- | --- | --- | --- | --- | --- | --- | --- |
| Mandyam (ARIC), 2013 | Prospective | US | European | 14538 | Adults sampled from 4 US communities | Female ≥ 460; Male ≥ 450 | New-onset AF | 19.7 yrs |
| Mandyam (CHS), 2013 | Prospective | US | European | 4745 | Adults recruited from the Medicare eligibility lists of 4 US countries and an additional 687 African Americans | Female ≥ 460; Male ≥ 450 | New-onset AF | 14.0 yrs |
| Mandyam (Health ABC), 2013 | Prospective | US | European | 2396 | Adults sampled from 2 US regions | Female ≥ 460; Male ≥ 450 | New-onset AF | 7.0 yrs |
| Nielsen, 2013 | Prospective | Denmark | European | 281277 | All subjects who underwent ECG recording at the Copenhagen General Practitioners’ Laboratory | ≥ 483 | New-onset AF | 5.7 yrs |
| Liu, 2015 | Retrospective | China | Asian | 242 | Patients with paroxysmal AF refractory to antiarrhythmic drugs and those with hypertension who underwent first time pulmonary vein | Female ≥ 460; Male ≥ 440 | Recurrent AF | 17.0±9.4 mos |
| O’Neal, 2015 | Prospective | USA | European | 6305 | Participants with no history of clinically apparent cardiovascular disease and no evidence of AF | Female ≥ 460; Male ≥ 450 | New-onset AF | 8.5 yrs |
| Wen, 2015 | Prospective | China | Asian | 39 | HCM patients undergoing their primary catheter ablation of symptomatic, drug-resistant AF | > 448 | Recurrent AF | 14.8 mos |
| Ma, 2016 | Prospective | China | Asian | 134 | Patients undergoing their first ablation with drug-refractory symptomatic PAF | > 440 | Recurrent AF | 29.1±12.4 mos |
| Wen, 2019 | Prospective | China | Asian | 120 | All HCM patients underwent primary catheter ablation | QTc > 448ms | Recurrent AF | 13.4 mos |
| Min, S, C, 2020 | Prospective | Koreans | Asian | 16793 | People underwent  medical check-ups at Asan Medical Center | > 450ms in men, > 470ms in women | New-onset AF | 10.0 yrs |
| Geurts, S, 2023 | Prospective | Netherlands | European | 12212 | participants free of AF | NA | New-onset AF | 9.3 yrs |

| **Cohorts, Year** | **Age, yrs** | **Female, %** | **AF Incidence, %** | **DM, %** | **HTN, %** | **CHD, %** | **HF, %** | **LVH, %** | **Medications** |
| --- | --- | --- | --- | --- | --- | --- | --- | --- | --- |
| Mandyam (ARIC), 2013 | 54.0±5.7 | 56.0 | 10.0 | 12.0 | 34.0 | 4.2 | 4.4 | NA | NA |
| Mandyam (CHS), 2013 | 72.0±5.5 | 59.0 | 27.0 | 15.0 | 57.0 | 17.0 | 2.6 | NA | NA |
| Mandyam (Health ABC), 2013 | 74.0±2.8 | 53.0 | 23.0 | 14.0 | 58.0 | 19.0 | 2.7 | NA | NA |
| Nielsen, 2013 | 54.0 | 56.0 | 4.0 | 6.0 | 16.0 | 2.0 | 1.0 | 4.0 | QTc Interval-prolonging drugs |
| Liu, 2015 | 62.9±8.9 | 40.0 | 59.5 | 8.7 | 100.0 | 14.0 | NA | NA | β-Blocker, Statin, ACEI or ARB, Ca2+ channel antagonist |
| O’Neal, 2015 | 62.0±10.0 | 54.0 | 4.4 | 13.8 | NA | NA | NA | 3.4 | Antihypertensive medications, Statins, Aspirin, Lipid-lowering medications |
| Wen, 2015 | 54.0±10.1 | 25.6 | 59.0 | NA | 0 | NA | 0 | 100.0 | NA |
| Ma, 2016 | 62.6±8.4 | 41.8 | 45.5 | 100.0 | 60.4 | 26.1 | NA | NA | Oral hypoglycemic agents, Insulin |
| Wen, 2019 | 57.4±10.7 | 35.9 | 57.5 | 15.0 | 35.0 | 15.0 | 9.2 | NA | warfarin or rivaroxaban or dabigatran |
| Min, S, C, 2020 | 48.2±9.4 | 37.7 | 2.0 | 5.9 | 13.9 | NA | NA | NA | NA |
| Geurts, S, 2023 | 64.9±9.6 | 58.2 | 10.5 | 10.2 | 59.2 | 6.2 | 1.7 | 6.2 | Cardiac, Antihypertensive, Beta blockers, Calcium blockers, Lipid lowering |

| **Cohorts, Year** | **HR (Bazett’s formula)** | **95%CI** | **p value** | **HR (Linear regression  model)** | **95%CI** | **p value** | **HR (every 10-ms prolongation)** | **95%CI** | **p value** |
| --- | --- | --- | --- | --- | --- | --- | --- | --- | --- |
| Mandyam (ARIC), 2013 | 1.56 | 1.22-2.01 | <0.001 | 1.99 | 1.37-2.89 | <0.001 | 1.11 | 1.07-1.14 | p<0.001 |
| Mandyam (CHS), 2013 | 1.29 | 1.01-1.66 | 0.045 | 1.57 | 1.18-2.07 | 0.002 |  |  |  |
| Mandyam (Health ABC), 2013 | 1.30 | 1.002-1.69 | 0.048 | 1.42 | 1.003-2.02 | 0.048 |  |  |  |
| Nielsen, 2013 | 1.74 | 1.45-2.09 | NA | 1.44 | 1.24-1.66 | <0.001 |  |  |  |
| Liu, 2015 | 2.767 | 1.661-4.611 | <0.001 |  |  |  | 1.156 | 1.037-1.29 | 0.009 |
| O’Neal, 2015 | 1.40 | 0.89-2.4 | 0.13 | 1.70 | 1.10-2.63 | 0.0023 | 1.10 | 0.97-1.2 | 0.130 |
| Wen, 2015 | 1.02 | 1.004-1.036 | 0.013 |  |  |  | 1.227 | 1.053-1.431 | 0.009 |
| Ma, 2016 | 1.026 | 1.012-1.04 | 0.005 |  |  |  | 1.286 | 1.039-1.591 | 0.021 |
| Wen, 2019 | 1.982 | 1.155-3.402 | 0.013 |  |  |  |  |  |  |
| Min, S, C, 2020 | 2.3 | 1.64-3.21 | <0.001 |  |  |  | 1.120 | 1.07-1.17 | <0.001 |
| Geurts, S, 2023 |  |  |  | 5.23 | 2.18-12.45 | 0.0002 |  |  |  |

**Abbreviations**: CHD: coronary heart disease; CI: confidence interval; DM: diabetes mellitus; HF: heart failure; HR: hazard ratios; HTN: hypertension; LAD: left atrial diameter; LVH: left ventricular hypertrophy; NA: not available; QTc: corrected QT interval.

**Supplementary Table S2** Subgroup analysis of the association between prolonged QTc interval and AF incidence risk based on Bazett’s formula.

| **Subgroup** | **Study, n** | **I^2^** | **P-Value of heterogeneity** | **HR** | **95%CI** |
| --- | --- | --- | --- | --- | --- |
| Type of AF | New-onset, 6 | 53.70% | 0.056 | 1.56 | 1.33-1.84 |
|  | Recurrent, 4 | 85.50% | <0.001 | 1.04 | 0.99-1.08 |
| Cut-off value | Female ≥ 460ms; Male ≥ 450ms, 4 | 0.00% | 0.702 | 1.38 | 1.20-1.59 |
|  | Others, 6 | 93.30% | <0.001 | 1.17 | 1.09-1.27 |
| Mean age | ＞60 yrs, 5 | 82.10% | <0.001 | 1.36 | 1.05-1.76 |
|  | ≤ 60 yrs, 5 | 94.40% | <0.001 | 1.62 | 1.12-2.33 |
| Population | European, 5 | 24.00% | 0.261 | 1.49 | 1.30-1.7 |
|  | Asian, 5 | 90.70% | <0.001 | 1.08 | 1.01-1.15 |
| Follow-up duration | < 5 yrs, 4 | 85.50% | <0.001 | 1.04 | 0.99-1.08 |
|  | ≥ 5 yrs, 6 | 53.70% | 0.056 | 1.56 | 1.33-1.84 |
| Location | Community, 6 | 53.70% | 0.056 | 1.56 | 1.33-1.84 |
|  | Hospital, 4 | 85.50% | <0.001 | 1.04 | 0.99-1.08 |

**Abbreviations**: CI: confidence interval; HR: hazard ratios.

**Supplementary Table S3** The sensitivity analysis for the association of prolongation QTc and AF recurrence.

| **The removed study** | **Study design** | **Year** | **Country or Institution** | **Ethnicity** | **Sample, n** | **OR** | **95% CI** | **P value** | **I^2^** |
| --- | --- | --- | --- | --- | --- | --- | --- | --- | --- |
| *Liu et al.* | Retrospective | 2015 | China | Asian | 242 | 1.024 | 1.001-1.049 | 0.043 | 67.00% |
| *Wen et al.* | Prospective | 2015 | China | Asian | 39 | 1.715 | 0.861-3.416 | 0.125 | 90.10% |
| *Ma et al.* | Prospective | 2016 | China | Asian | 134 | 1.712 | 0.855-3.426 | 0.129 | 90.20% |
| *Wen et al.* | Prospective | 2019 | China | Asian | 120 | 1.029 | 0.990-1.069 | 0.149 | 86.60% |
| Pooled estimate |  |  |  |  | 535 | 1.035 | 0.990-1.082 | 0.127 | 85.50% |
